# Community monitoring of coliform pollution in Lake Tanganyika

**DOI:** 10.1371/journal.pone.0262881

**Published:** 2022-01-28

**Authors:** Happiness Anold Moshi, Daniel Abel Shilla, Ismael Aaron Kimirei, Catherine O’ Reilly, Wim Clymans, Isabel Bishop, Steven Arthur Loiselle

**Affiliations:** 1 Tanzania Fisheries Research Institute, Kigoma Centre, Kigoma, Tanzania; 2 Department of Aquatic Sciences and Fisheries Technology, University of Dar es Salaam, Dar es Salaam, Tanzania; 3 Tanzania Fisheries Research Institute, Dar es Salaam Headquarters, Dar es Salaam, Tanzania; 4 Department of Geography, Geology and the Environment, Illinois State University, Normal, IL, United States of America; 5 VITO, Antwerp, Belgium; 6 Earthwatch Europe, Oxford, United Kingdom; 7 University of Siena, Siena, Italy; INRA/Sorbonne University, FRANCE

## Abstract

Conventional water quality monitoring has been done for decades in Lake Tanganyika, under different national and international programs. However, these projects utilized monitoring approaches, which were temporally limited, labour intensive and costly. This study examines the use of citizen science to monitor the dynamics of coliform concentrations in Lake Tanganyika as a complementary method to statutory and project-focused measurements. Persons in five coastal communities (Kibirizi, Ilagala, Karago, Ujiji and Gombe) were trained and monitored total coliforms, faecal coliforms and turbidity for one year on a monthly basis, in parallel with professional scientists. A standardized and calibrated Secchi tube was used at the same time to determine turbidity. Results indicate that total and faecal coliform concentrations determined by citizen scientists correlated well to those determined by professional scientists. Furthermore, citizen scientist-based turbidity values were shown to provide a potential indicator for high FC and TC concentrations. As a simple tiered approach to identify increased coliform loads, trained local citizen scientists could use low-cost turbidity measurements with follow up sampling and analysis for coliforms, to inform their communities and regulatory bodies of high risk conditions, as well as to validate local mitigation actions. By comparing the spatial and temporal dynamics of coliform concentrations to local conditions of infrastructure, population, precipitation and hydrology in the 15 sites (3 sites per community) over 12 months, potential drivers of coliform pollution in these communities were identified, largely related to precipitation dynamics and the land use.

## Introduction

Since the beginning of 21^st^ century, many developing countries including Tanzania have experienced rapid urbanization, increasing economic transformation, industrialization and population growth. This has led to an increased pressure on water bodies which receive direct and indirect discharges of wastewater [1], resulting in severe water challenges to local and regional communities [2, 3].

Water quality monitoring is a fundamental tool for the management of lakes, rivers, estuaries, wetlands, and marine systems. With urbanization and agricultural activities impacting waterbodies at an unprecedented level in many African countries, there is a clear need for more information about spatial and temporal dynamics of ecosystem conditions. Such information is fundamental to understand the drivers and identify mitigation strategies necessary to conserve these ecosystems [4, 5].

Microbial and pathogenic pollution can jeopardize public health when lake surface waters are used for drinking, agricultural and recreational uses [6]. A common indicator for assessing water quality for human health is the estimation of coliform bacteria concentrations. However, the determination of coliform concentrations are usually performed by trained scientists in the laboratory [7]. The use of optical approaches can be used as an early indicator of changes in contaminant load, including microbial contamination. Turbidity measurements, for example, are very easy to perform and require minimal training and equipment [8].

Although coliform bacteria are non-pathogenic bacteria that occur in faeces of warm blooded animals, their presence in elevated concentrations has been correlated with various waterborne diseases such as gastrointestinal illness, typhoid fever, ear infections, diarrhoea and dysentery [9]. Presence of coliform bacteria in surface water should at least be seen as a probable threat or indicator of deterioration of microbiological water quality [10] and implies that enteric pathogens may also be present [11]. Coliform bacteria are more numerous, and relatively easy to determine in a laboratory, making them reliable indicators of faecal pollution [12].

Lake Tanganyika, like many African water bodies, is experiencing an increase in pollution loads from local sources. This is particularly true in the small coastal towns in the Kigoma municipality on the north-east shore of the lake, where most lack integrated wastewater treatment systems [12]. Sanitation is limited to pit latrines, pour flush latrines, flushing toilets with septic tanks with a limited incidence of open defecation. Pit latrines are not built for structural strength, but for privacy, and some direct their wastes into Lake Tanganyika. Some members of the fishing community living near the lake defecate directly into Lake Tanganyika offshore [13]. This results in multiple direct health risks to the coastal communities as well as a loss of ecosystem services as local economic activities (eg. fishing and farming) are reliant on lake water and lake waters are used for washing, fishing or drinking [2].

Conventional water quality monitoring has been done for years in Lake Tanganyika under different national and international programs [14–21], that have generated new and important information about lake dynamics and critical tipping points, including the FAO/FINNIDA Lake Tanganyika Research project (1993–1998), the GEF/World Bank Lake Tanganyika Biodiversity Research project (1997–2001), Climate variability in Lake Tanganyika CLIMLake and CLIMFISH (BELSPO) (2002–2006), and the DANIDA Projection of climate change effects in Lake Tanganyika (CLEAT) (2014–2019). However, these projects utilized monitoring approaches that were typically temporally limited, labour intensive and often requiring boats to collect water samples, which were then transported, to the laboratory for analysis. Because such monitoring cannot be easily maintained outside of the scope of these projects, water quality monitoring in Lake Tanganyika has been intermittent and inconsistent. There is a need to explore monitoring techniques that complement existing datasets, can be supported on a long-term basis, and, crucially, can be used locally to inform management actions to improve coastal water quality [22].

A potentially complementary method to provide information on the dynamics of the coastal waters is citizen science [23], a technique that has been used to great extent in monitoring aquatic environments in many parts of the world [24–27]. Citizen science is the partnering of scientists with volunteers to answer scientific questions, reducing costs associated with research projects and creating a more comprehensive data collection [28]. Trained citizen scientists can produce data that is comparable to professionals for a variety of parameters and habitats [29, 30]. Citizen science can also enhance community understanding and awareness of environmental issues, improve conservation efforts, natural resource management and environmental protection [31]. Water quality monitoring has been a particular focus for citizen scientists worldwide, with well-established initiatives like ‘FreshWater Watch’ providing data that can contribute to local, national, and international policy and management decisions [32]. However, most of these initiatives focus on water chemistry and turbidity, which require limited training and relatively inexpensive equipment [33]. Far fewer citizen science studies measure the microbiological contamination due to the cost and complexity of the methods used.

Microbial pollution can have important impacts on local communities in the African Great Lakes [34]. Importantly, microbial pollution events in small coastal communities are often directly related to the activities and local infrastructure that are controlled by the very communities that are impacted. However, without the possibility to monitor these conditions, focused mitigation approaches cannot be applied and validated. In this study, we explore the potential for citizen science to contribute to the monitoring and management of faecal pollution in the freshwater ecosystems that they regularly use for multiple purposes. Specifically, we develop and test a citizen science-based approach for determining changes in coliform concentrations affecting communities on the shores of Lake Tanganyika, Tanzania. Finally, we explore potential drivers related to wastewater management, population growth and land use change and their links to increased pollution periods.

## Material and methods

### Study design

This study used a longitudinal design with measurements made throughout the year (from May 2019 to April 2020) to explore the spatial and temporal dynamics of coliform and turbidity. Measurements were taken by trained citizen scientists and professional scientists from Tanzania Fisheries Research Institute, Kigoma centre. A total number of 180 water samples were collected and measured by each group of citizen scientists and professional scientists.

### Study area

**Fig 1:**
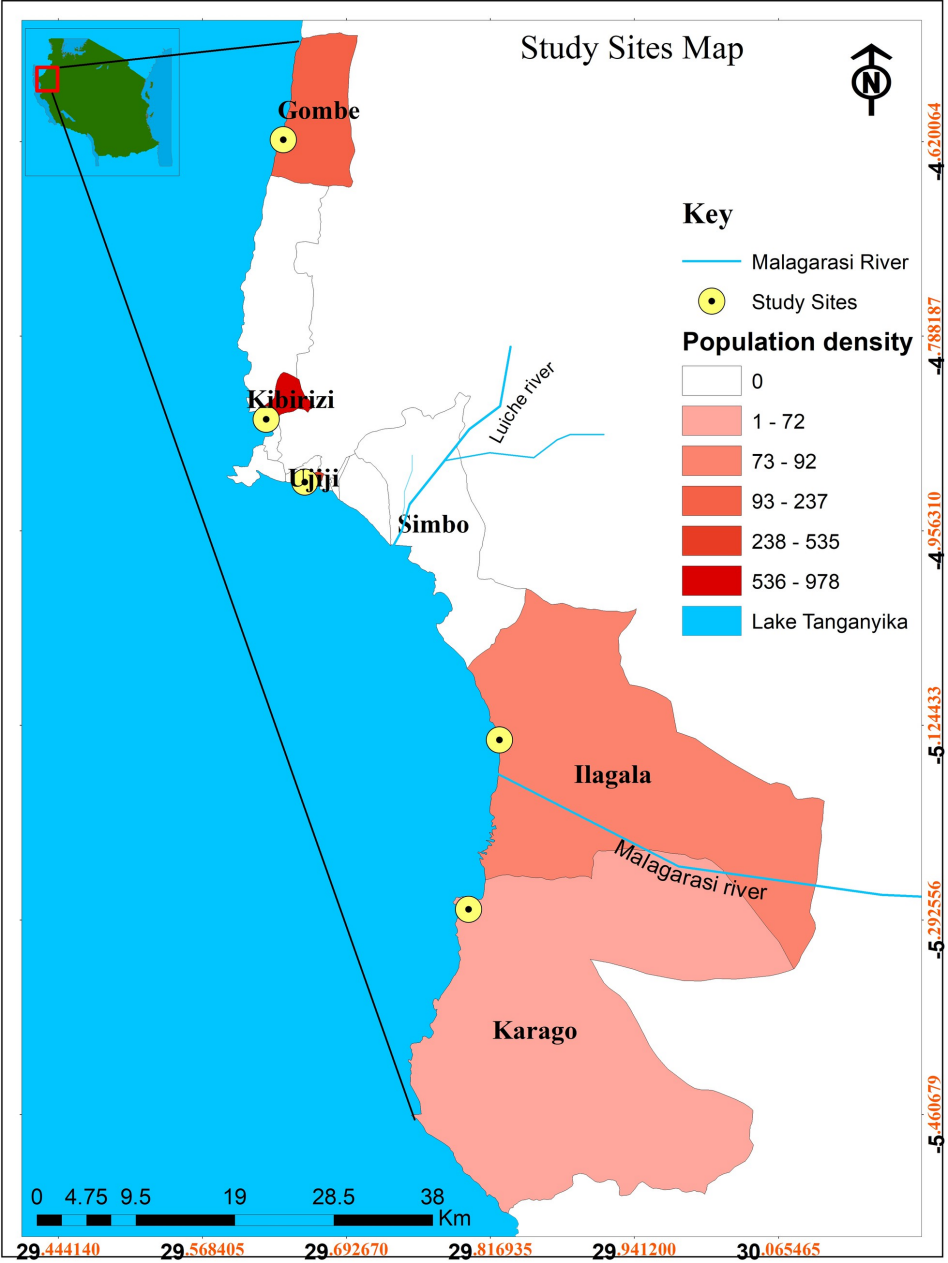
A map of Lake Tanganyika showing study sites.

Water samples were collected from lake surface waters in five coastal communities with different levels of environmental and economic pressures. All communities were in the Kigoma municipality and along Lake Tanganyika (Tanzania side): Gombe, Kibirizi, Ujiji, Ilagala and Karago (Fig 1). Three sub-sites were chosen in each community (Table 1). Each measurement site was located between 4 and 1700m away from the lake shores and were chosen based on their different distance from the shore, population size and potential sources of microbiological pollution. Ease of access by professional scientists and citizen scientists was also considered.

**Table 1:** Location and description of study sites and associated sub-sites.

Site	Sub-site	Coordinates	Distance from the shore (m)	Description
Kibirizi	Kibirizi 1	-4.8611S, 29.6272E	6	Peri-urban site located at 3-4km from Kigoma town, with a total area of 12.5 km^2^ and a population of 12,225. Municipal discharge in the area.
	Kibirizi 2	-4.8630S, 29.6286E	9	
	Kibirizi 3	-4.8650S, 29.6233E	1200	
Ujiji	Ujiji 1	-4.9244S, 29.6752E	7	Peri-urban site located 8-10km from Kigoma town with an area of 10.2 km^2^ and a total population of 9040. The area is very close to Luiche river mouth and receives emissions from it. Farming activities take place.
	Ujiji 2	-4.9180S, 29.6622E	15	
	Ujiji 3	-4.9244S, 29.7063E	6	
Ilagala	Ilagala 1	-5.2119S, 29.8422E	4	Peri- urban site with an area of 231.8 km^2^ and a total population of 21,246 This site is very close to Malagarasi river mouth and is impacted by its emissions. Farming activities take place
	Ilagala 2	-5.2116S, 29.8436E	13	
	Ilagala 3	-5.1552S, 29.8261E	6	
Karago	Karago 1	-5.2813S, 29.7969E	12	Rural site with an area of 75.7 km^2^ and a population of 5456. This site is in a closed bay and receives effluents from Malagarasi river.
	Karago 2	-5.2877S, 29.7988E	22	
	Karago 3	-5.2855S, 29.7894E	1700	
Gombe	Gombe 1	-4.6269S, 29.5183E	4	Located within protected national park with an area of 22.3 km^2^ and a population of 5270. It is surrounded by forest.
	Gombe 2	-4.6344S, 29.6316E	5	
	Gombe 3	-4.6411S, 29.6297E	10	

### Recruitment and training of citizen science for water quality monitoring

A total of 25 participants, five from each community were recruited from people holding at least Tanzania certificate of secondary education (ordinary level) and older than 18 years of age. Equal opportunity was provided to all qualified participants regardless of gender during the entire recruitment process. Each participant was given a short screening survey to assess their interest in aquatic sciences, environment issues and willingness to participate in lake water quality monitoring. Out of 25 participants, 10 participants (2 in each community) were identified as the potential citizen scientists based on their interest, availability and willingness to participate in lake water quality monitoring (S1 Table). All potential citizen scientists underwent a standard field training and safety course using FreshwaterWatch training materials which include theoretical and hands-on experience on microbial and turbidity measurements. The training was divided into theoretical and practical sessions. For the theoretical session, the training was conducted for two days (16 hours) while the practical session was conducted for 5 days (40 hours). During training, each citizen scientist was required to collect and measure samples of water for total coliform, faecal coliform and turbidity to confirm that they were sufficiently trained and they can participate in the monitoring. Each individual citizen scientist was provided an airtime voucher (for Internet and cell phone services) of 5000 Tanzania shillings (~ €1.85) for each sampling month. Feedback regarding the results and their significance of the measurements was provided to the citizen scientists by the participating researchers. The citizen scientists then transmitted this information to the local communities.

### Coliform monitoring

Surface water (0–30 cm) samples for bacterial analysis were collected from each selected sub-site located randomly from 4 to 1700m away from the lake shore (Table 1), following standard sampling guidelines procedures [35]. Both trained citizen scientists and professional scientists from Tanzania Fisheries Research Institute performed identical sample incubation and counting procedures on paired samples over the course of the study. In order to avoid error during sampling, the water samples in each sub-site for citizen scientists and professional scientists were collected in the same bucket and then each group removed the required samples and performed the sample preparation independently on two replicate samples. Each sample was kept in sterilized borosilicate glass stoppered bottles (250 mL), stored in the dark between 6–10°C and transported to the laboratory within 24 hours of sampling.

The membrane filtration method was used for analysis and counting of coliform colony forming units (CFU) per each 100 mL of water sample. Samples were filtered through a 47 mm cellulose nitrate membrane filter, with a 0.45μm pore size. The bacteria trapped on the surface of the filter was then carefully removed and placed in a sterile petri dish containing the solidified media and incubated for 24 hours at 37°C and 44.5°C for total coliforms and faecal coliforms, respectively.

The M-FC Media used for faecal coliform bacteria was prepared by both Tanzania Fisheries Research Institute professional scientists and trained citizen scientists, using Tryptose 10.0 g, Proteose peptone 5.0g, Yeast extract 3.0 g, Sodium Chloride 5.0 g, Lactose 12.5 g, Bile Salts mixture 3 1.5 g, Aniline blue 0.1 g, Agar 15.0 g and 1000 mL of water. The M- ENDO Media used for total coliform bacteria was prepared by both Tanzania fisheries research institute professional scientists and citizen scientists using Tryptone 3.7 g, Peptone 3.7 g, Tryptose 7.5 g, Yeast extract 1.2 g, Lactose 9.4 g, Sodium Chloride 3.7 g, Dipotassium Hydrogen Phosphate 3.30 g, Potassium Dihydrogen Phosphate 1.0 g, Sodium Lauryl Sulphate 0.05 g, Sodium Deoxycholate 0.10 g, Sodium Sulphite 1.6 g, Basic Fuchsin 0.80 g, Agar 15.0 g and 1000 mL of water.

Replicate samples from each site (n = 15) were averaged for each month, providing 180 paired samples (citizen scientist and professional) over one year for comparison and analysis.

### Turbidity monitoring

Water samples for turbidity measurement were collected in each sub-site by trained citizen scientists using a clean plastic bottle. After rinsing, a standard and pre-calibrated Secchi tube was used in situ to determine turbidity in nephelometric turbidity units (NTU). Citizen scientists were trained to slowly fill the tube with the water sample until the Secchi disc at the bottom of the tube was no longer visible. The Secchi tube allowed for measurements of turbidity between 14 to 240 NTU [36]. Single measurements were made monthly at each site, allowing for 180 measurements of turbidity of the year.

### Rainfall

Lake Tanganyika lies within the east African rift valley and is characterized by a four to five-month cool dry (∼25 °C) season from May to September and a warm (∼28 °C) wet season from October to April. Kigoma receives a mean annual rainfall of about 935 mm and a monthly mean of 36.5 mm [37]. Precipitation data were obtained from Tanzania Meteorological Agency (Kigoma station) which is located approximately 7.2 Km from the lakeshore.

### Population and activity drivers

According to the 2012 national census, the regional population is 2,127,930 over an area of 45,066 km^2^, with the highest population densities occurring on the lake shore. The population data for each study site was acquired from the Tanzania National Bureau of Statistics 2012 population and housing census [38].

### Land use

In the Kigoma area, natural forest makes up the largest land cover of 45.2%, grazing area of 27.1%, 17.8% waterbodies and 9.9% for other activities, mostly farming. In the present analysis, the dominant land use was assigned as either urban or rural land cover for each site using Google maps and local observations.

Spatial differences in potential drivers related to land use, population and infrastructure in each site were considered, including village population, population density, percentage of households with toilets, distance of the site from households, level of economic activity, farming intensity, number of livestock, slope of the land with respect to the lake, closed or open bays and urban/rural dominance. These physical and socio-economic characteristics were estimated from available population and housing census data, with distance from households being that from the site to the nearest house. Binary values (1 and 0) were assigned by the authors for non-continuous data (economic activity, farming intensity, closed/open bays, urban/rural dominance) based on available data and personal observations. A value of 1 was given for high economic activity (active market or small industry) and 0 was assigned for low economic activity. A value of 1 was also given to high farming intensity (cultivation area greater than 30% of total area) and 0 for low farming intensity (cultivation area less than 30% of the total area [39]). Closed/open bays, urban/rural dominance were based on visual comparisons using Google Maps. Ordinal values were given to continuous values such as rural/urban dominance, closed/open bay (Table 2).

**Table 2:** Characteristics of the five study sites.

Study site	Population density^a^	% of Toilets^a^	Number of Animals (livestock)^a^	Slope from the land to the lake/1000^b^	Closed bay/open bay^c^	Farming intensity^c^	Total population^a^	Distance from households (m) to the site^b^	Economic activity^d^	Rural/Urban dominance^c^
Karago	72	75	4321	1	semi-closed	0	5456	507	0	Rural
Ilagala	92	90	22,653	22	open	1	18087	333	1	Urban
Gombe	237	80	7,120	60	open	0	5270	533	0	Rural
Kibirizi	978	80	21,181	1	semi-closed	0	12225	347	1	Urban
Ujiji	535	85	19,992	10	open	1	9040	240	1	Peri-Urban

^a^ Data obtained from Tanzania population and housing census (2012),

^b^ calculated from direct measurements,

^c^ values from Google Maps and

^d^ estimated from direct observations.

### Statistical analysis

Data were analysed using standard methods, including paired t-Test (two-paired sample for means), one-way Analysis of Variance (ANOVA), Pearson and Spearman correlations. Multiple regression was performed to understand the significance of potential factors influencing coliform concentration, including: population, population density, farming intensity, economic activity, slope of the land to the lake, households with toilets, number of livestock and precipitation with total and faecal coliform concentration as dependent factors. All data were tested with an alpha level of significance of 0.05 and using a Bonferroni correction for multiple correlations using Realstats (version 2016). Measurements and data collection were not impacted by Covid-19 restrictions.

## Results

### Temporal dynamics of coliform concentrations and turbidity

**Fig 2:**
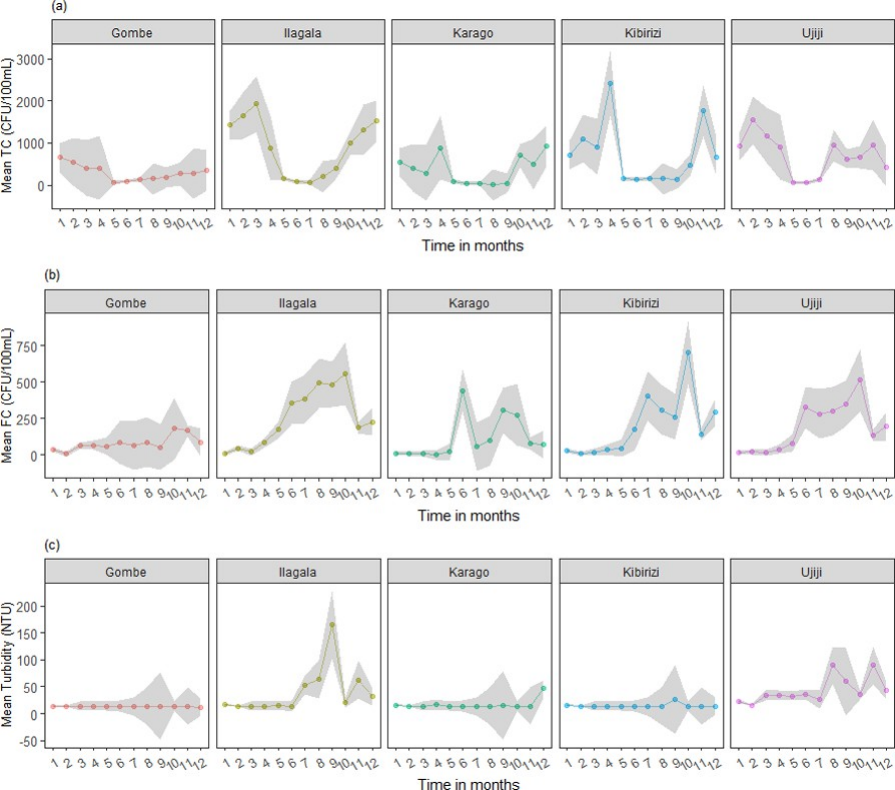
Monthly mean concentrations (in line) ± standard deviation (shaded area) of coliforms and turbidity determined by citizen scientists in each study site. (a) Mean total coliform counts (CFU/100 mL) ± standard deviation, (b) Mean fecal coliform count (CFU/100 mL) ± standard deviation (c) Mean turbidity (NTU) ± standard deviation.

Total coliform concentrations showed a strong seasonal dynamic, with higher concentrations measured in the rainy season (905 CFU/100 mL) compared to the dry season (171 CFU/100 mL) (t = 9.5, p < 0.001). A similar dynamic was observed for faecal coliforms, with a lower t-statistic, but following the same trend with higher rainy season concentrations (288 CFU/100 mL) compared to the dry season (114 CFU/100 mL) (t = 4.6, p < 0.001). Study sites differed in their seasonal coliform dynamics (Fig 2A and 2B). Turbidity also showed a seasonal behaviour, with the rainy season having a higher mean (32 NTU) with respect to the dry season (17 NTU) (t = 3.0, p = 0.003) (Fig 2C).

**Fig 3:**
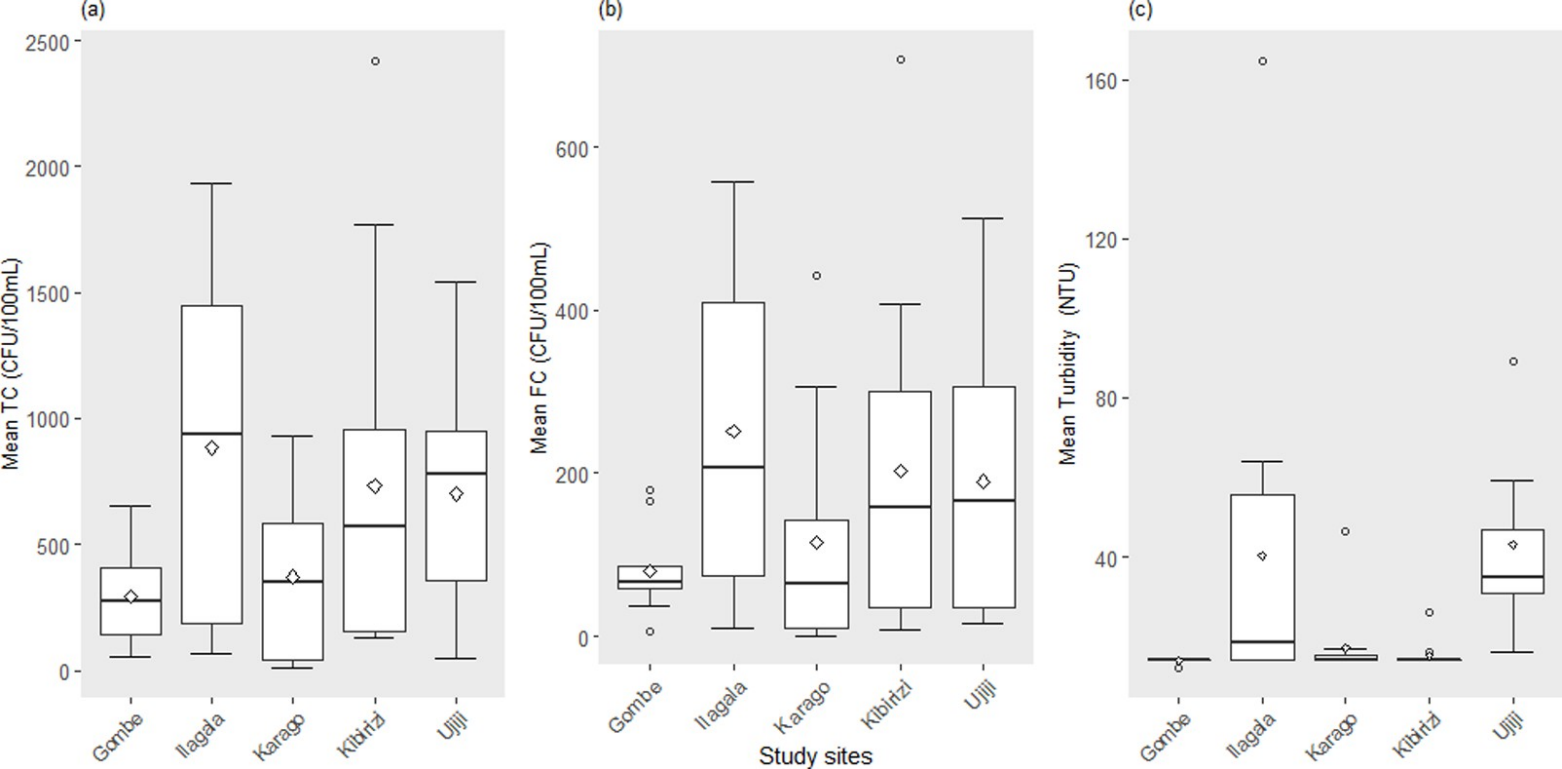
Boxplot representing mean concentrations ± standard deviation of coliform and turbidity determined by citizen scientists in 5 study sites (n = 36 per site). (a) Mean total coliform counts (CFU/100 mL) (b) Mean fecal coliform counts (CFU/100mL) (c) Mean Turbidity (NTU). Bars represent standard deviation and points represent variation around mean.

Spatial Dynamics of Coliform and TurbidityThere was a clear difference in the average total coliform concentration between sampling sites (ANOVA, df = 4, F = 5.2, p <0.001; Table 3). The TC counts were highest at Ilagala and lowest at Gombe and Karago, with an overall average of 568±246 CFU/100mL, considering all sampling sites. Likewise, there was a significant difference in faecal coliform among sites (df = 4, F = 6.7, p <0.001; Fig 3B). Ujiji displayed the highest FC counts with the lowest at Gombe and Karago. The overall average of all sampling sites was 188±88 CFU/100mL. Similarly, turbidity concentrations were significantly different among site (df = 4, F = 5.4, p = 0.003; Fig 3C), with the highest turbidity at Ilagala and the lowest again at Gombe.

**Table 3:** Average and standard deviation of coliform and turbidity for each study site (n = 36).

Average concentrations	Gombe	Ilagala	Karago	Kibirizi	Ujiji
Faecal coliforms (CFU/100 mL)	80 ± 70	251±231	115±184	203 ±274	287 ± 323
Total coliforms (CFU/100 mL)	255 ±271	835±816	364 ±359	677± 907	708 ± 551
Turbidity (NTU)	14±0	42±57	17±11	15 ± 6	38 ± 54

### Potential drivers for coliform variabilities

Multiple linear regression showed low effect size for total coliforms and faecal coliforms (r^2^ = 0.31 and 0.17, p < 0.001), with the only significant coefficient being that related to monthly average precipitation. The regression with turbidity (r^2^ = 0.11, p = 0.02) had significant coefficients for monthly average precipitation as well as the relative distance of the site from households.

By comparing concentrations with respect to local conditions, economic activity level, distance of the site from household, farming intensity, river impact and urban or rural dominance, it was possible to identify which site attributes were linked to higher coliform or turbidity conditions. Significant differences (T-test, p < 0.05, Table 4) in average total coliform concentrations were found for sites with: higher economic activity, shorter distance from households, higher farming intensity, river proximity and urban dominance. Significant difference in average faecal coliform concentrations were found for sites with: higher economic activity, higher farming intensity, river proximity and urban dominance. Higher turbidity was associated with sites with higher economic activity, shorter distance from households, higher farming intensity, river proximity and urban dominance.

**Table 4:** t-Test: Paired two sample for means showing the monthly mean ± Standard Deviation (S.D) of potential drivers influencing concentration of coliform and turbidity in the study sites separated by different characteristic, t-static and p-value at 0.05. The variables which were significant are bolded.

Land use	Total coliform (CFU/100mL)			Faecal coliform (CFU/100mL)			Turbidity		
	Mean ± S.D	t-static	p-value	Mean ± S.D	t-static	p-value	Mean ± S.D	t-static	p-value
High Economic activity	772.7 ± 534	4.06	**0.0009**	214.9 ± 183.5	3.04	**0.005**	32.8 ± 20.9	2.7	**0.01**
Low Economic activity	332.9 ± 239			97.5 ± 84.4			15.5 ± 4.3		
Close to household	717 ± 527.3	-1.91	**0.04**	196.5 ± 181.7	-1.7	0.1	29.1 ± 12.4	-1.5	0.1
Far from household	516.6 ± 366			148.8 ± 113.7			23.7 ± 14.7		
High farming intensity	792.7 ±534	-2.89	**0.007**	220.8 ± 178.9	-3.6	**0.001**	41.7 ± 30.1	3.07	**0.01**
Low farming intensity	466 ± 370.9			132.7 ± 112.6			15.3 ± 3		
River impacted	792.7 ± 534	-2.89	**0.007**	220.8 ± 178.9	-3.6	**0.001**	41.7 ± 30.1	-3.07	**0.01**
Non-river impacted	466 ± 370.9			132.7 ± 112.6			15.3 ± 3		
Urban	772.6 ± 534	4.06	**0.0009**	214.9 ± 183.5	3.04	**0.01**	32.8 ± 20.9	2.7	**0.01**
Rural	332.9 ± 239			97.5 ± 84.4			15.4 ± 4.3		

Seasonal precipitation dynamics were seen as a potential driver of coliform and turbidity dynamics. We compared monthly precipitation (total mm/month) to coliform and turbidity averages for each study site (Pearson correlation, Table 5). Faecal coliforms showed the highest correlation with precipitation in Ilagala and Kibirizi, while the correlations were much poorer for the other sites, indicating that the dynamics of these latter sites were not well associated to those of monthly precipitation. Total coliform dynamics showed a better correlation with precipitation, with significant correlations with monthly precipitation dynamics in all sites except Ujiji. Monthly dynamics for turbidity were most similar to monthly precipitation in Ujiji and Ilagala.

**Table 5:** Comparison of t-scores related to Pearson correlations comparing monthly dynamics of coliform and turbidity with monthly dynamics of precipitation for each study site.

T-score for Pearson correlations:	Gombe	Ilagala	Karago	Kibirizi	Ujiji
Average monthly faecal coliforms	1.94	3.00	0.94	2.04	0.12
Average monthly total coliforms	3.25	5.65	3.89	3.45	1.89
Average monthly turbidity	1.33	2.05	1.03	0.55	2.93
Critical T-score (df = 10)	2.23	2.23	2.23	2.23	2.23

### Relationship between turbidity and coliforms

**Fig 4:**
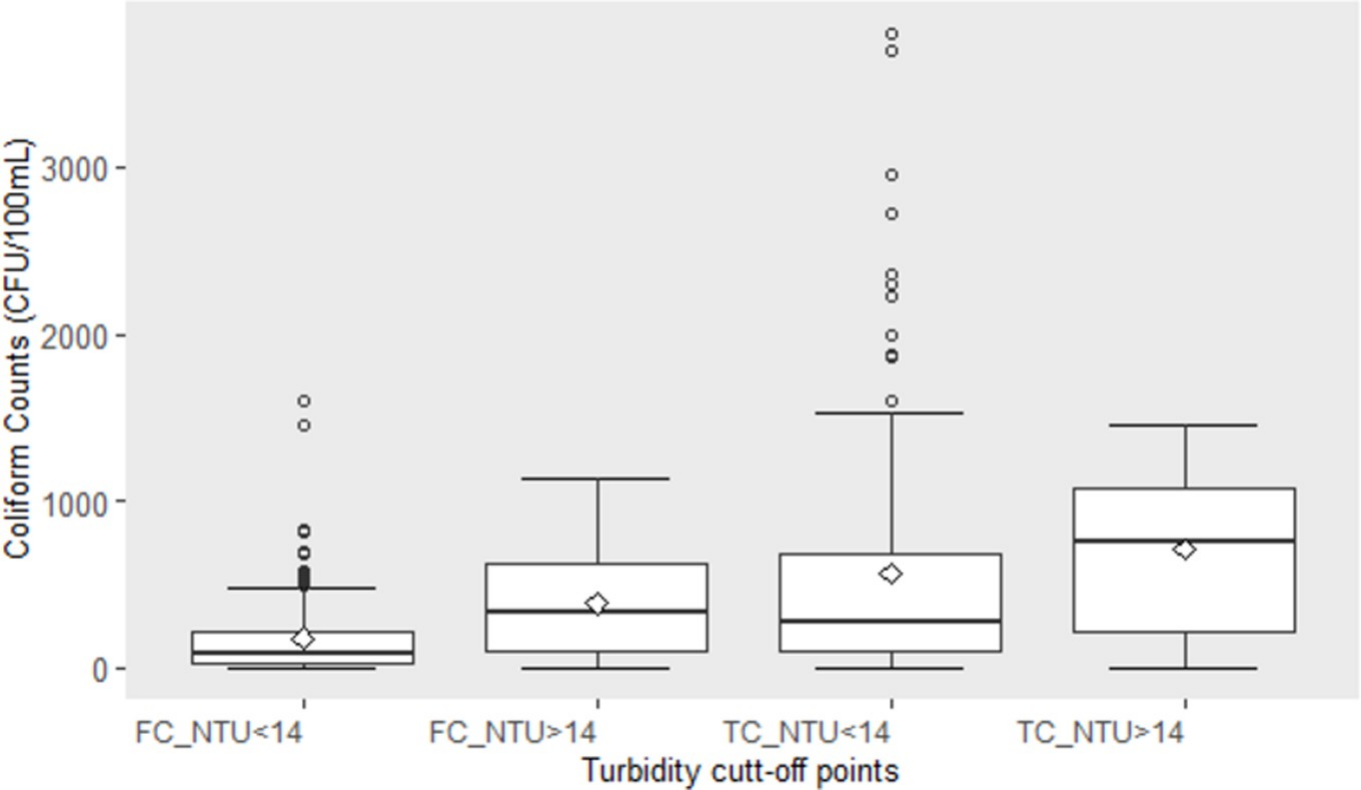
Boxplots of Mean coliform counts (CFU/100mL) determined by citizen scientists based on turbidity measurements, above and below 14 NTU cutoff points. FC_NTU<14 = Mean fecal coliform counts below 14 NTU, FC_NTU<14 = Mean fecal coliform counts above 14 NTU, TC_NTU<14 = Mean total coliform counts below 14 NTU and TC_NTU>14 = Mean total coliform counts above 14 NTU. Bars represent standard deviation and points represent variation around mean.

While the temporal dynamics of monthly averaged coliform and turbidity followed a similar trend (Fig 2), there was no significance correlation between these individual measurements (r < 0.10, p > 0.05). However, when the coliform data were divided into measurements taken in conditions of high turbidity (> 14 NTU) and low turbidity (*≤* 14 NTU), clear differences emerged. The mean and median concentrations of faecal coliforms were significantly different for the two turbidity categories (Fig 4) (T-test, p < 0.001). For total coliforms, there was no significant difference.

Considering a turbidity cut-off of 14 NTU, it was possible to evaluate whether Secchi tube measurements could be a proxy for local conditions of high coliform concentrations. The 14 NTU cut-off was associated to a depth of 433 mm or 95% of the total length of a calibrated Secchi tube. When turbidity is below 14 NTU, there was a 76% probability of faecal coliform concentration to be below 200 CFU/100mL. For the same turbidity cut-off, there was a 70% probability that total coliform counts were below 600 CFU/100mL. Turbidity along the coastal area of Lake Tanganyika can be highly variable and is strongly influenced by the presence of rivers and coastal activities [51], with open lake values ranging from 2 to 21 NTU [52].

### Comparison of citizen and professional scientist measurements

**Fig 5:**
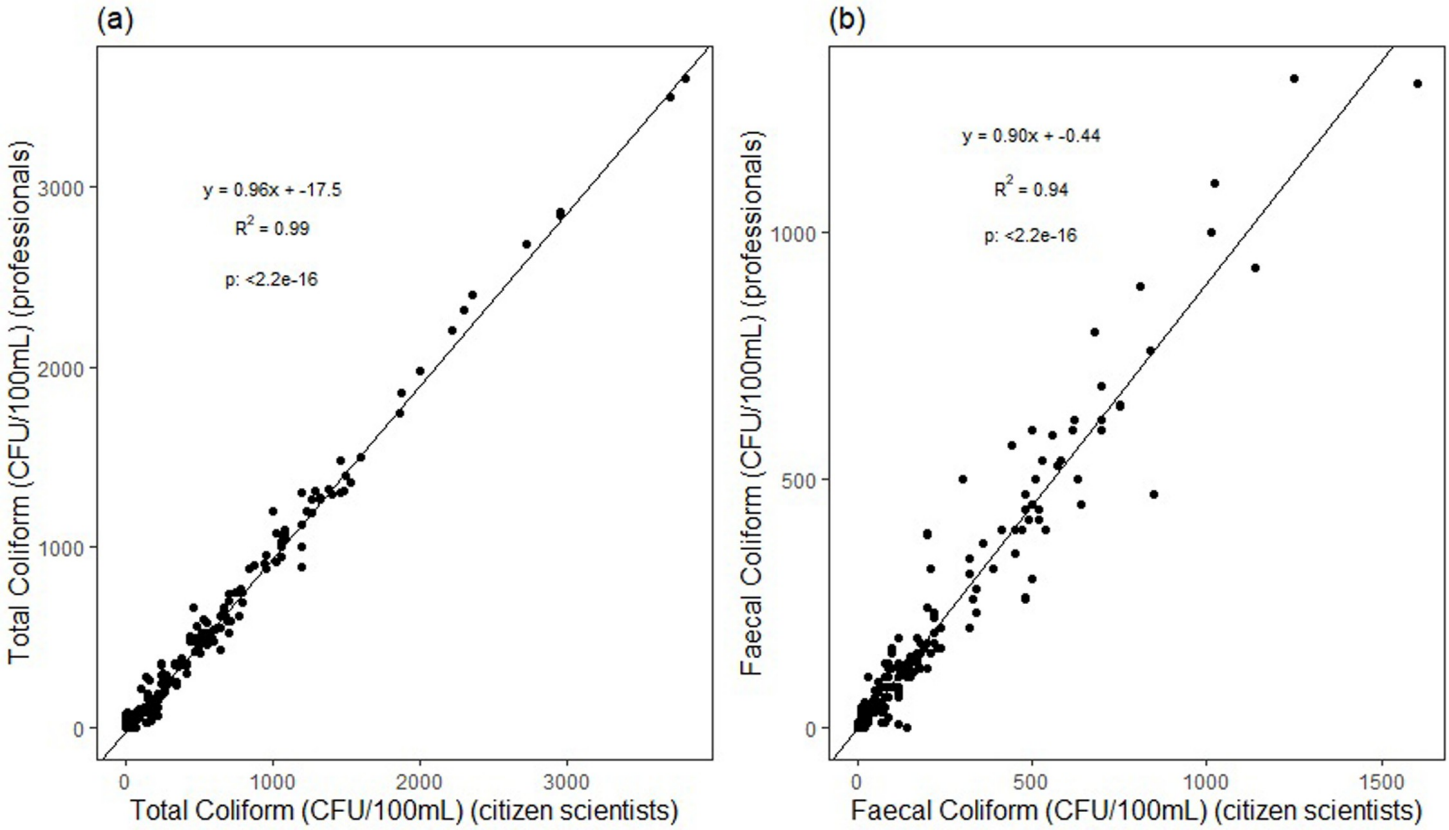
Pearson correlation of total coliform and fecal coliform concentration (CFU/100 mL) observed by professional and citizen scientists (a) Total coliform (CFU/100 mL) (b) fecal coliform (CFU /100m/L) (n = 180).

The correlation for total coliforms between professional scientists and citizen scientists was very strong (Spearman rho = 0.99, n = 180) (Fig 5A), while the regression equation showed a minor overestimation of citizen scientists’ values over professional scientists’ values; (professional scientists CFU = 0.96*citizen scientists CFU– 17.5 CFU, p <0.001).

The correlation for faecal coliforms between professional scientists and citizen scientists was also very strong (Spearman rho = 0.94, n = 180) (Fig 5B), while the regression equation showed a slightly larger overestimation of citizen scientists’ values with respect to professional scientists values; (professional scientists CFU = 0.90*citizen scientists CFU– 0.44 CFU, p <0.001).

## Discussion

Faecal contamination of near shore waters in many African lakes is a major challenge for local communities and national public health agencies, as people in the coastal communities often use lake water for cooking and washing. The concentrations observed in the present study are similar to those measured in Lake Malawi, with coliform concentrations of 35-400CFU/100mL in nearshore surface waters [40], and lower than those reported for Lake Victoria’s fish landing sites (3.6×10^6^ CFU/mL, [41] and Lake Victoria’s Nakivubo channel (up to 9.2×10^6^ CFU/mL, [42]. In the Burundian surface waters of Lake Tanganyika, concentrations are similarly elevated, averaging 33250CFU/100mL and 2000CFU/100mL for total and faecal coliforms, respectively [1]; similar to our study, population density seems to be a major driver.

In the present study, Ilagala, Kibirizi and Ujiji presented the highest coliform concentrations, as well as a high sensitivity to precipitation. Ilagala has a high population and receives effluents from Malagarasi river, the second largest river in Tanzania. The effluent load becomes particularly elevated in the rainy season when the river can overflow. Kibirizi has an important fishery industry, with the largest fish market among the studied sites and related number of visitors. Kibirizi is also located in a closed bay, with a higher retention of runoff. While not receiving any discharge from any major river, Kibirizi receives waters from temporal streams which flow into the lake during the rainy season. These streams drain surrounding areas which have small population centres as well as agricultural activities.

Gombe and Karago presented the best conditions, with the lowest coliform concentrations and turbidity of the study sites. Gombe is a small community, located near a national park where most economic activities are prohibited. Most of the area around Gombe is mountainous and well vegetated, which reduces possible influx of livestock and human wastes. Karago is a small rural area in a semi-closed bay, with a low population and animal density. Karago showed no significant linkage between faecal coliform and precipitation dynamics, suggesting that large quantities of wastewater do not flow directly into the lake during heavy rain events. Both sites had low economic and agricultural activities, leading to a low use of animal wastes and other pollutants that might increase contamination and particle concentrations in the nearby lake waters.

Ujiji presents mixed conditions, with high coliforms and turbidity, but a low influence from precipitation. In fact, Ujiji has the lowest relative monthly variance of total and faecal coliforms of any of the study sites, indicating a more regular emission and a lower sensitivity to seasonal patterns in climate. Ujiji has a population similar to that Ilagala and is also located near the discharge of river. The Luiche River drains in Ujiji area and several agricultural villages. River borne particulate and dissolved matter from agricultural activities, grazing and other anthropogenic activities can be significant [43, 44]. The combination of close proximity of the houses and the influence of the Luiche River both lead to conditions of high coliform concentrations and high turbidity in Ujiji, with limited seasonal variation.

The comparative study of coliform concentrations measured by trained and equipped citizen scientists to those determined by professional scientists show a very high correlation, with a small overestimation for both total and faecal coliforms. This suggests that citizen scientists, well trained and equipped, can make accurate estimates of coliform concentrations that could be used to complement municipal and national monitoring efforts. Sampling in periods of high risk (eg. rainy season) or local community citizen scientists in collaboration with laboratories in Kigoma could perform suspected moments of high coliforms. Interestingly, measurements of high turbidity provided information on conditions of elevated coliform loads, allowing for the creation of a tiered approach to monitoring coastal lake conditions and informing local communities of high risk conditions.

The use of turbidity to estimate loads of coliform bacteria is attractive due to the rapidity with which turbidity can be determined automatically [45] through in situ sensors [46] or by drone [47] or satellite sensing [48, 49]. In the present study, manual measurements of turbidity were made by trained citizen scientists using a simple calibrated Secchi tube. A turbidity cut-off of 14 NTU provided good accuracy for separating periods of low and high faecal and total coliform concentrations. This implies that when turbidity is higher than 14 NTU, there is an elevated possibility of coliform pollution, which warrants further testing or possibly, community-based restrictions on lake water use until conditions improve.

During and after rain events, surface runoff increases the possibility that dissolved and particulate matter from agricultural areas and human settlements are carried into the lake. Studies have shown that coliform dynamics in surface waters can be strongly modified by local precipitation [50–52]. In Lake Victoria, higher concentrations of microbial pollutants in the wet season have been associated to storm water run-offs from multiple sources [53] while studies in different climate zones have found similar results [54–56]. It should be noted that coastal conditions can be highly variable after rain events, showing the importance of replicate sampling during the rainy season [7, 57]. Our study was limited to regularly monthly sampling and not focused specifically on the rain events.

The concentration of coliform across the sampling sites was also found to be influenced by farming intensity, number of toilets, population, number of animals and rural versus urban dominated land uses (Table 2). Anthropogenic factors, such as land use and wastewater treatment have often been associated to elevated coliform concentrations [56, 58], as have agricultural activities requiring the use or storage of animal manure [59]. Further study would be required to identify the specific links between different land use types and coliform contamination in lake waters to develop appropriate mitigation measures [60]. This kind of information could be gained from the use of similar citizen science methods across more coastal communities of Lake Tanganyika.

## Conclusions

According to Tanzania guidelines for drinking water [61], the permissible faecal coliform for drinking water is less than 1 CFU/100 mL. There were no measurements (n = 180) taken over the 12- months period where this standard was met. A more appropriate standard is the WHO guideline for permissible faecal coliform counts for recreational purposes of 200 CFU/100 mL or that for irrigation purposes of 1000 CFU/100 mL [62]. Using the former, community citizen scientists along the shores of Lake Tanganyika could use calibrated standard Secchi tubes to identify conditions where lake water is no longer appropriate for direct use (eg. washing) without further treatment. Using a tiered approach, a first screening result of high turbidity could be followed by further testing for coliforms, reducing the costs and challenges related to the training and equipment necessary for coliform sampling and identification. Developing a sampling strategy based on lower cost approaches can represent a powerful tool to monitor changes in water quality when applied at high frequency [63]. There is a clear opportunity for local populations to obtain high frequency data using a $2 Secchi tube when regulatory monitoring by professional technicians is limited [64]. With developments in simple optical tools for smartphones, there is a growing opportunity for rapid assessment of lake conditions by community members. Citizen science programmes have costs related to recruiting, training and equiping volunteers, creating an open database that can receive monitoring data and perform associated quality control, as well as feedback and exchange with active participants. These costs will depend on the number of participants and availability of local scientists.

These results suggest that communities can contribute to monitoring their local waters for microbiological conditions. Similar successes by citizen scientists monitoring benthic communities [65], phytoplankton concentrations [66] and nutrient pollution [30] have been reported. Given that the infrastructure for coastal communities is unlikely to change over the short term, the results of the present study indicate that community-based monitoring, with trained local citizen scientists, could inform municipal and national agencies as well as those same communities of poor water quality conditions as well as validate the efficacy of mitigation actions and management strategies initiated by the same community.

## Supporting information

S1 Table Demographic characteristics of citizen scientists participated in coliform and turbidity monitoring in Lake Tanganyika. (DOCX)

S1 Graphical abstract (TIF)
