# Supplementary material for: Community monitoring of coliform pollution in Lake Tanganyika
Source: PLoS One. 2022 Jan 28;17(1):e0262881. doi: 10.1371/journal.pone.0262881 (PMC8797266; doi:10.1371/journal.pone.0262881)
Supplement: S1 Table — (DOCX) [file pone.0262881.s001.docx]

S1 Table: Demographic characteristics of citizen scientists participated in coliform and turbidity monitoring in Lake Tanganyika.

| Village Name | Age | Gender | Education | Economic activity | Residence time in the village | Willingness to participate in coliform monitoring |
| --- | --- | --- | --- | --- | --- | --- |
| Kibirizi | 34 | F | Secondary education | Fish processing | 34 | Yes |
|  | 40 | M | Secondary education | Fishing | 24 | Yes |
| Ujiji | 23 | M | Secondary education | Fishing | 23 | Yes |
|  | 39 | M | Secondary education | Teaching | 30 | Yes |
| Ilagala | 32 | F | Secondary education | Clinical laboratory officer | 32 | Yes |
|  | 39 | M | Secondary education | Farming | 39 | Yes |
| Karago | 28 | F | Secondary education | Farming | 12 | Yes |
|  | 32 | M | Secondary education | Fishing | 32 | Yes |
| Gombe | 35 | M | Secondary education | Fishing | 35 | Yes |
|  | 21 | M | Secondary education | Fish processing | 21 | Yes |
