# Supplementary figures and images for: Community monitoring of coliform pollution in Lake Tanganyika
Source: PLoS One. 2022 Jan 28;17(1):e0262881. doi: 10.1371/journal.pone.0262881 (PMC8797266; doi:10.1371/journal.pone.0262881)

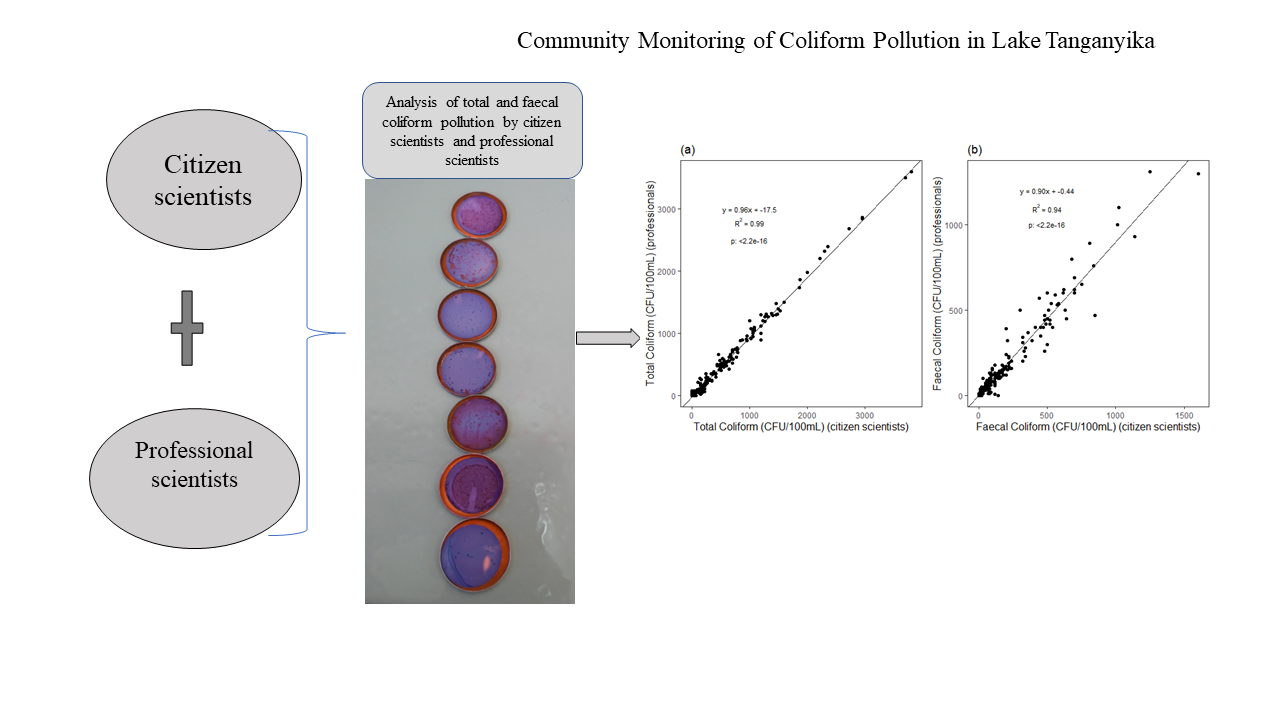

Supplement: S1 Graphical abstract — (TIF) [file pone.0262881.s002.tif]
